# A Report of the Facts and Circumstances Relating to a Case of Compound Fracture and Prosecution for Mal-Practice, in Which William Smith Was Plaintiff, and Drs. Goodyear and Hyde Defendants, at Cortland Village, Cortland County, N. Y. March, 1841: Comprising Statements of the Case by Several Medical Gentlemen, Together with Notes and Comments on the Testimony

**Published:** 1842-02

**Authors:** 


ART. V.—*A Report of the Facts and Circumstances relating to a case of Compound Fracture and Prosecution for Mal-Practice, in which William Smith was Plaintiff, and Drs. Goodyear and Hyde Defendants, at Cortland Village, Cortland County, N. Y. March* 1841: *Comprising statements of the case by several medical gentlemen, together with Notes and Comments on the Testimony.* By A. B. SHIPMAN, M. D. Cortlandville, 1841. pp. 35.

The following extracts from the history of this case, as given by Dr. Shipman in the pamphlet, will put the readers of the Journal in possession of the principal matters involved.

"On the 4th of July, 1839, Wm. Smith fell from a building in consequence of the staging giving way, and fractured his leg. I was called upon to visit him, and arrived about two hours after the accident—found both bones broken, tibia two inches above the ankle joint, fibula 4 inches. The end of the tibia protruded through a laceration in the soft parts on the inside of the leg, 4 inches long, constituting what is called in surgery a compound fracture. None of the important blood vessels or nerves were injured. The fractured end of the tibia which protruded was transverse on the inside, and a small portion of its diameter on its outside, or next the fibula, was detached, and had fallen out. The extreme end of the bone was denuded of its periosteum, perhaps one fourth of an inch. One small spicula of loose bone was removed from the wound with the fingers. No dirt or foreign bodies were in the wound, nor was there any considerable hemorrhage. After sponging the wound, extension and counter extension was made, and the bones placed in apposition. Narrow strips of adhesive plaster, bringing the wound accurately together, were applied—Scultetus' bandage, and two long splints well padded, reaching above the knee and below the ankle, with another short splint along the front of the leg—these were secured by strong tapes, and the leg extended upon a pillow, with directions to keep it constantly wet with cold spirits and water. An anodyne of sulphate of morphine was administered, and I left him. The patient was about 50 years of age, of a strong and robust constitution, but addicted to intemperance."

On the following day, July 5th, Mr. Smith was removed to the Alms-House. Dr. Shipman's attendance then ceased, and he heard no more of the case until the thirteenth, when he was requested to repair to the Alms-House to assist in amputating Smith's leg, Drs. Goodyear and Hyde, the medical officers of the establishment, having given it as their opinion that this must be done immediately.

"On arriving at the Alms House, Drs. Goodyear and Hyde were there with some others, and we were soon joined by Dr. Lewis Riggs, G. W. Bradford, and Joel R. Carpenter—Wm. J. Wilson, Benton and Maybury, Medical students, were also present. Two of the Superintendents, Messrs. Coye and Allen, were there and present at the consultation. After examining the limb, the medical gentlemen retired to a private room for consultation. Instead of proceeding to amputate the limb, as the nature of the summons had prepared us to expect, the question arose as to the propriety of amputating at all. The patient at this time was in the following condition:—His leg lay over the double inclined plane, the bone protruding through the wound nearly two inches: it was dry and dark, and had lost its vitality—thə foot was turned off and the leg distorted, with retraction of the muscles and shortening of the limb; the leg was swollen and inflamed nearly to the knee; the wound gaped, but there was no loss of substance; upper part of the wound had healed, and healthy granulations covered a portion of the bone; some pus of good quality issued from beneath the bone. The patient suffered much pain, yet was free from fever; appetite, strength, and pulse good; tongue clean, and bowels regular. Drs. Goodyear and Hyde urged the necessity of immediate ampuation. The reasons they assigned, were, the age, habits, constitution of the patient, state of the weather, and apprehensions of fever. Dr. Bradford thought it a case in which the propriety of amputation might be "*talked of.*" Drs. Riggs, Carpenter, and myself, saw no reasons why amputation was required: there were neither local nor constitutional symptoms which demanded it."

" I advised the removal of the dead portion of bone with the saw, which offered an obstacle to the reduction of the fracture, placing the bones in apposition, and applying appropriate dressings; that it was important that the fractured ends of the fibula be placed in apposition, that it might unite, which would serve to keep the limb steady until union of the other bone had taken place—to give such constitutional remedies as the case required. and I was confident the leg might be saved without endangering the life of the patient."

* * * "The defendants did not relish this advice; they urged the necessity of immediate amputation, for fear, as they expressed it, "*that a fever might set in and carry the patient off*"—*that the age, habits of the patient, and state of the weather*, were other reasons why amputation should be performed. I knew of no authority in Surgery where age, habits, state of the weather, and *apprehensions of fever* were sufficient reasons for removing a limb. Drs. Riggs and Carpenter coincided with me in opinion, advised the removal of the bone, and dressing with splints. Late in the afternoon I returned home. I saw no more of the case until the 23d of July, ten days after the consultation. On Sunday the 22d, a messenger came to my house and informed me that the Superintendents of the Poor had given Smith liberty to choose his surgeon, and he had sent for me. Monday morning 23d, I met Dr. Carpenter at the Alms House. Found the patient in nearly the same condition as on the day of consultation—the limb rather more distorted; bone still protruding. The heel was very sore from pressure upon the inclined plane, and had sloughed to the bone. *The same kind of dressing, a loose cloth, was upon the limb.* He complained of much pain in his heel and leg, especially on motion or a jar of his bed. With the assistance of Dr. Carpenter I proceeded to remove the end of the bone. A retractor was placed under it, and the lower end held by a pair of strong forceps, and the leg supported by an assistant—about an inch of the end was removed by the amputating saw, including that which had lost its vitality. After the removal of the bone, the limb was placed in an easy position and left. At the Alms House I learned, that Dr. Patterson, of Homer, had been also requested to assist in taking charge of the patient, and we concluded to defer the application of splints until the next day. July 24th met Drs. Patterson and Carpenter; applied the proper dressings; cleaned the wound from pus and maggots; brought the edges as near together as possible with adhesive plaster; removed the double inclined plane; applied splints; dressed the heel; placed the limb upon a pillow in the straight position; ordered him quinine, stimulants and anodynes. From this time Drs. Patterson, Carpenter, or myself, saw him daily and dressed the wound, keeping it clean and the bone in place. The wound kept on healing and contracting steadily and progressively, without any bad symptoms ensuing, until union took place, first in the fibula, which served to keep the leg steady without trouble; the tibia next united, and the patient left his bed and went upon crutches. He remained in the Alms House during the winter, but left in the spring and engaged in labor. His leg has become strong, and he walks without difficulty and without much lameness."

In February 1841, a suit was instituted by Mr. Smith against Drs. Goodyear and Hyde for mal-practice, and the trial came on in March following. To support the prosecution, he introduced as witnesses Messrs. Brockway and Rose, Drs. Shipman, Riggs, Patterson and Carpenter, Mr. Allen, Superintendant of the Poor, and Baker, nurse at the Alms-House. The statement of the case given above, is in accordance with the testimony of these persons. The defendants, on the other hand, endeavored to show that they had treated the case faithfully and correctly, that amputation of the leg would have been proper, and, collaterally, that the excision of the dead portion of bone, as advised and performed by Dr. Shipman and others, was censurable. To this end they adduced the evidence of Drs. Bradford, Webster (late Professor of Surgery, and now of Anatomy, in Geneva Medical College), Hamilton (Professor of Surgery in Geneva College, and likewise in two or three other colleges we believe), Hawley Maybury, Benton, Webster, Burdick and Smith, and that of Mr. Seymour, keeper of the poor-house. After the examination of the witnesses for both parties, the suit was withdrawn by the plaintiff's counsel.

In the treatment of the case, the reader would at once suppose there had been culpable negligence on the part of the medical officers of the poor-house, did they not take pains to show that they visited the patient "day after day," and that the limb was regularly dressed by them. At the same time it is not attempted to be concealed, that the fragments passed each other; that "one end of the bone stuck out through the hole (in the soft parts) shortly after the first dressing, and remained so till the council and so after, till Shipman dressed it;" and that the foot lay off at an angle of ten or twelve degrees. It reduces itself then to culpable igorance; and that any professional man, much less a professor of surgery and a professor of anatomy in a reputable school, could be found to approve such treatment, passes our understanding. Nothing *could* be proper that would allow such a state of things to exist, in a transverse fracture of the leg, for 19 or 20 days! Argument is useless; the bare fact is sufficient.

Neither is argument necessary in reference to the question of amputation. How such a design could have been for a moment entertained, we cannot possibly conceive. The local symptoms could not justify it: there was some pain, and the bone had lost its vitality for the space of an inch; but the discharge from the wound was healthy, and granulations had formed over a portion of its surface. The constitutional symptoms did not call for it; on the contrary, the general health was not much deranged: there was no loss of appetite, the tongue was clean, the bowels in good condition; the strength was well-sustained, and the pulse was good. Are men's limbs to be sacrificed to "*apprehensions* of fever?" The idea is ridiculous. In the absence of every other motive, one might almost suppose that amputation was desired to get rid of a troublesome case, and the more effectually to conceal a bad piece of surgery.

The removal of the necrosed portion of bone was clearly the most obvious remedy; and we think any one who reads the statement of the case given above, must assent to its propriety. It could be effected without any difficulty. Nature, in fact, which is so frequently mentioned by defendant's witnesses, seems to have pointed out this very course. There was the extremity of the tibia, as it were offering itself to the saw! there were healthy granulations, both on the upper and lower part of the wound—in the latter point concealing the lower fragment—all showing conclusively that nature, with a little assistance, was ready and competent to repair the injury! To have waited for exfoliation, as was proposed, would have doomed the patient to incalculable pain and a long confinement; whilst the severe local irritation, combined with the profuse discharge attending the process, reacting on the constitution, would in all probability have finally rendered amputation necessary.*

A portion of the pamphlet is devoted to remarks and animadversions on the testimony of some of the defendant's witnesses. This testimony, to which we have already alluded incidentally, certainly exhibits some curious matters. That of Professor Webster strikes us as being of a most singular character. We quote some passages: "In this case the amount of irritation would have prevented me from any effort to this effect," viz. cutting off the protruded bone. "I should have adjusted the bones and reduced irritation, and watched the period when nature was incompetent to do her duty, then I might be disposed to amputate." . . . "Taking every thing into consideration, I should have decided on amputation." "It is of no importance that the foot fell over because the bones could not unite in such a case." . . . "I do not know of any thing wrong in this case." "It is not good practice to allow a fracture to get out of place—it indicates want of skill." It is to be expected that there will be contradiction in the testimony adduced by opposite parties, but it is certainly unusual for a witness thus to contradict in one breath what he has said in another.

Not less obnoxious is the evidence of Professor Hamilton. He commences with the assertion, that "the man stands an *equal chance* of losing his limb," although there was a perfect bony union, and the leg had entirely recovered, save a small sinus which continued to discharge! No doubt there would have been quite as much propriety in removing it then, as there was at the time of the consultation. He goes on to say: "The probable cause of the death of the bone, was the injury the periosteum received: the mere inflammation of the periosteum will cause the bone to die." . . . "If the external cover-

---

* Or another result might have been the formation of callus, fixing the bones in their unnatural position. The reader will find under the head of "Selections," in this number of the Journal, a case, similar in many respects to Smith's, in which this termination occurred, requiring subsequently a painful and tedious operation to correct the deformity.

ing was off, the bone must exfoliate." "As to the cutting off the bone, there was but one circumstance under which it was proper, and that is where we suppose or presume that the living parts are dying by lying in contact with the dead; the rule is the same as to amputation." . . . "As to moving the foot, if I understand the testimony, it did not matter a straw." . . . . "After the first dressing, I could not have treated the case better than defendants did." It is greatly to be feared that the multiplied duties of this gentleman (we have already said he was Professor of Surgery in four different medical schools) have prevented him from keeping pace with the progress of surgical knowledge. If such be the doctrines taught by him and his colleague (Professor Webster), suits for mal-practice will excite no wonder in that section of country, nor, for that matter, would suits for *mal-teaching*.

After perusing statements like the above from men whose position and opportunities have given them every advantage, the reader will be less surprised at the following from those whose sphere of observation has been more confined, and some of whom in all likelihood received their medical education at the hands of the gentleman above-named. Dr. Hawley testifies very sagely as follows: "The turning off the foot and projection of the bone made but little difference for the first two or three weeks, until ossific union began." "In the case in question, should not have hesitated to amputate; at the time of consultation should have amputated." . . . . "I know of no authority that would warrant the cutting off the bone in this case, at the time." . . . "Have seen the limb; not certain but it will be amputated yet!" Another witness held forth in this wise: "It was no serious injury for twenty days to allow the bone to get out of place." "As to cutting the bone off the time it was done, should suppose it would be an injury to it; the result does not prove it good practice at all." "In this case the limb is not better than a wooden one." And another, as if to go beyond all who had preceded him, affirms that it was "not material to keep the bones in apposition at all; as to the fibula, not material; confining it would have tended to mortification;" and that "the result proves that amputation would have been right!"

The perusal of this "Report" has given us a favorable impression both of the talents and professional skill of the author. Its publication seems to have been a measure of self-defence merely, and to have been reluctantly undertaken. Soon after the trial, the most gross mis-statements of the case were published and distributed among the adjoining counties, and rumors were circulated in every direction, prejudicial to the character of Dr. Shipman, and of the professional gentleman associated with him in the treatment. In commenting on the testimony of some of the witnesses for the defence, he speaks as any man would who felt himself unjustly censured, though, we confess, in much milder terms than we should have spoken under similar circumstances. The result of the case must be peculiarly gratifying to him, at the same time that it is a triumphant refutation of all the slander and falsehood of which he has been the subject. At the date of the publication, the leg was free from ulcer or discharge of any kind, and in all respects nearly as serviceable as the other. C.

